# Quantitative Evaluation of Microcirculatory Alterations in Patients with COVID-19 and Bacterial Septic Shock through Remote Photoplethysmography and Automated Capillary Refill Time Analysis

**DOI:** 10.3390/medicina60101680

**Published:** 2024-10-14

**Authors:** Mara Klibus, Darja Smirnova, Zbignevs Marcinkevics, Uldis Rubins, Andris Grabovskis, Indulis Vanags, Olegs Sabelnikovs

**Affiliations:** 1 Department of Clinical Skills and Medical Technology, Rīga Stradiņš University, LV-1007 Riga, Latvia; mara.klibus@rsu.com (M.K.); darja.smirnova@rsu.lv (D.S.); 2 Department of Anaesthesiology and Reanimatology, Rīga Stradiņš University, LV-1007 Riga, Latvia; indulis.vanags@rsu.lv; 3 Department of Anaesthesiology and Reanimatology, Pauls Stradins CUH, LV-1002 Riga, Latvia; 4 Faculty of Medicine and Life Sciences, University of Latvia, LV-1063 Riga, Latvia; zbigis@latnet.lv; 5 Faculty of Science and Technology, University of Latvia, LV-1063 Riga, Latvia; uldisrubins@yahoo.com (U.R.); andris.grabovskis@gmail.com (A.G.)

**Keywords:** microcirculation, septic shock, remote photoplethysmography, automated capillary refill time

## Abstract

*Background and Objectives:* Sepsis, a leading global health challenge, accounts for around 20% of deaths worldwide. The complexity of sepsis, especially the difference between bacterial and viral etiologies, requires an effective assessment of microcirculation during resuscitation. This study aimed to evaluate the impact of infusion therapy on microcirculation in patients with sepsis, focusing on bacterial- and COVID-19-associated sepsis using remote photoplethysmography (rPPG) and the automated capillary refill time (aCRT). *Materials and Methods:* This single-center prospective study was conducted in the ICU of Pauls Stradins Clinical University Hospital, including 20 patients with sepsis/septic shock. The patients were selected based on hemodynamic instability and divided into COVID-19 and Bacterial Septic Shock groups. Fluid responsiveness was assessed using the Passive Leg Raising Test (PLRT). Systemic hemodynamics and microcirculation were monitored through MAP CRT, rPPG, and serum lactate levels. Statistical analyses compared responses within and between the groups across different stages of the protocol. *Results:* The Bacterial group exhibited higher initial serum lactate levels and more pronounced microcirculatory dysfunction than the COVID-19 group. rPPG was more sensitive in detecting perfusion changes, showing significant differences between the groups. The automated CRT demonstrated greater sensitivity compared to the manual CRT, revealing significant differences during PLRT stages between bacterial- and COVID-19-associated sepsis. Both groups had a transient hemodynamic response to PLRT, with subsequent stabilization upon fluid infusion. *Conclusions:* When managing patients with sepsis in intensive care, monitoring microcirculation is of paramount importance in infusion therapy. Our study highlights the potential of rPPG and aCRT as tools for this purpose. These techniques can be used in conjunction with routine parameters, such as lactate levels and systemic hemodynamic parameters, to provide a comprehensive assessment of a patient’s condition.

## 1. Introduction

Sepsis, a significant global health challenge, contributes to around 20% of all-cause deaths worldwide, with mortality in intensive care patients being estimated to be between 30% and 40% [1,2,3,4]. This life-threatening condition arises from a dysregulated host response to infections, including bacterial, viral, or fungal origins. The complexity of sepsis is further emphasized by the varying pathophysiological impacts of different types of infections; for instance, bacterial sepsis typically involves systemic hypercoagulation and fibrinolysis, while severe viral infections like COVID-19 are associated with localized thrombus formation [5].

The hallmark of sepsis is the disturbance of microcirculation, leading to decreased oxygen delivery, tissue hypoxia, and organ dysfunction. Current resuscitation therapies in septic shock, focusing on fluid therapy and vasopressor support, aim to maintain systemic hemodynamics and counter tissue hypoperfusion [3,6]. However, these strategies do not always translate into effective microcirculatory restoration, potentially leading to complications like fluid overload [7].

The quest for an effective microcirculation assessment has led to the exploration of innovative methods such as remote photoplethysmography (rPPG) and automated capillary refill time (aCRT). These are complemented by techniques like near-infrared spectrometry (NIRS) and hand-held vital microscopes (HCMs), which facilitate bedside microcirculation monitoring. Despite their potential, these methods face challenges in routine clinical application [8,9,10]. Contrast-enhanced ultrasound (CEUS), employing gas microbubbles, has been notably used in patients with COVID-19 to assess lung perfusion anomalies [8,11].

Clinically, tissue peripheral perfusion evaluation involves using bedside parameters like the capillary refill time (CRT) and serum lactate levels. The CRT, a dynamic parameter, assesses the skin color return time post-pressure application, with recent trials like ANDROMEDA shock suggesting its role in guiding resuscitation strategies [12]. Additional indicators such as skin temperature and mottling score provide insights into organ dysfunction severity and patient prognosis [11,13]. The Peripheral Perfusion Index (PPI), indicating vascular tone, also offers critical information about microcirculatory changes during sepsis.

However, the manual CRT is subjective, and serum lactate levels lack specificity, underscoring the need for more comprehensive assessment parameters. These include the perfused capillary density, the proportion of perfused capillaries, the microvascular flow index, and the heterogeneity flow index, which are all crucial for understanding the interaction between perfusion and tissue metabolic demands.

This study aims to assess the effects of infusion therapy on microcirculation in patients with sepsis, focusing on the differences between bacterial and viral etiologies. By employing microcirculation assessment techniques such as rPPG and aCRT, we seek to investigate whether bacterial sepsis results in more significant microcirculatory changes compared to COVID-19-associated sepsis.

We hypothesize that bacterial sepsis induces more significant microcirculatory changes than COVID-19-associated sepsis, and that these changes can be detected by rPPG and aCRT techniques.

## 2. Materials and Methods

### 2.1. Study Design and Setting

This was a single-center, prospective study conducted at the general intensive care unit (ICU) of Pauls Stradins Clinical University Hospital (PSCUH). The research protocol received approval from the Riga Stradiņš University Ethical Committee (approval number PĒK-22-2/299/2021), adhering to the principles of the Declaration of Helsinki.

### 2.2. Patient Selection Criteria

This study included 20 patients aged 18 to 70 years who were admitted to the ICU with a diagnosis of severe pneumonia or/and Acute Respiratory Distress Syndrome (ARDS), requiring intensive care. Criteria for inclusion centered on hemodynamic instability, defined as a Mean Arterial Pressure (MAP) < 65 mmHg despite fluid resuscitation and vasopressor use. Fluid resuscitation commenced with crystalloids (normal saline or Ringer’s lactate) at 10 mL/kg/h. Vasopressors were administered according to institutional guidelines, and sedation was achieved using midazolam, fentanyl, and propofol. Exclusion criteria included patients without hemodynamic instability, those outside the specified age range, those who were pregnant, or those with severe cardiac or renal impairments (NYHA class IV heart failure, LVEF < 30%, GFR < 15 mL/min/1.73 m^2^, or stage 5 CKD).

### 2.3. Assessment of Fluid Responsiveness and Study Protocol

Fluid responsiveness was evaluated using the Passive Leg Raising Test (PLRT) [14]. A positive response was defined as an increase in pulse pressure of ≥10% as determined by bedside vital sign monitor [15]. Patients with a positive PLR test received additional crystalloid infusion at 10 mL/kg/h. The study protocol was organized into four stages: T1—a five-minute baseline assessment in the supine position; T2—PLRT with legs elevated to 45 degrees for 5 min; T3—post-PLRT with legs returned to the baseline position for 5 min; and T4—60 min following fluid infusion.

### 2.4. Group Stratification

Patients responding positively to PLRT were categorized into two groups:

COVID-19 Group: Ten patients with a positive SARS-CoV-2 PCR test, clinical signs of sepsis, and elevated inflammatory markers (WBC, PCT, and CRP) without secondary bacterial infection.

Bacterial Septic Shock Group: Ten patients with positive blood cultures, clinical signs of sepsis, and increased inflammatory markers.

### 2.5. Data Collection and Monitoring Techniques

During all stages (T1–T4) of the measurement protocol, macrohemodynamic and microcirculation characterizing parameters were collected from the patients.

#### 2.5.1. Systemic Hemodynamics Monitoring

Continuous evaluation of systemic hemodynamics involved measuring the mean arterial blood pressure (MAP) and recording vasopressor dosages. The MAP was tracked using a Philips Intelevue X3 MX750 bedside monitor(Philips, Eindhoven, The Netherlands) which was directly connected to an arterial blood pressure catheter for real-time monitoring. This allowed for precise adjustments in patient management based on dynamic hemodynamic changes. Vasopressor dosages were also documented from the infusion pumps, offering a comprehensive view of the pharmacological support provided to the patients.

#### 2.5.2. Microcirculation Assessment

Capillary refill time (CRT): CRT was measured through two distinct methods: manual (mCRT) [16] and automated (aCRT). For the mCRT, a trained investigator applied gentle pressure to the patient’s fingertip until the capillary bed blanched. Upon release, the time taken for the return of normal color was recorded with a chronometer. This traditional approach to assessing peripheral circulation was conducted at heart level to ensure physiological relevance. The aCRT was conducted using a novel custom prototype (Blazar Ltd., Riga, Latvia) with an optoelectronic system designed to provide a more standardized and objective measurement [17]. The device applied a uniform force (approximately 1 kg, equivalent to 410 mmHg pressure) and used a specific wavelength (525 nm) to detect blood volume changes, enabling the calculation of the total capillary refill time (TST). Both mCRT and aCRT measurements were repeated five times at each time point for accuracy and averaged for analysis. Details on the device operation are provided elsewhere [17].

#### 2.5.3. Remote Photoplethysmography (rPPG)

Peripheral perfusion was assessed non-invasively using rPPG [18]. This optical technique uses light reflection to detect changes in blood volume within the microvascular bed. The setup consists of a white LED light source (100 W electric power), an industrial camera (Ximea-xiQ USB-3.0 (XIMEA GmbH in Münster, Germany), ADC 8-12-bits, resolution 648 × 488 pix. with a mounted lens (Edmund Optics, C-mount f = 25 mm (Edmund Optics Ltd., Barrington, IL, USA), and a 540 nm CWL 10 nm FWHM narrow-band filter (Edmund Optics Ltd., Barrington, IL, USA). The dorsal aspect of a patient’s palm was placed at a fixed distance of 30 cm from the light source, and the reflected light signals, indicative of pulsating blood flow, were captured and analyzed. The perfusion index (PI), representing the relative strength of cutaneous blood perfusion, was calculated from the processed single period PPG signal as the ratio between the pulsatile blood flow (PPG signal AC component) to the non-pulsatile blood (PPG signal DC component) in peripheral tissue and recorded at each assessment period [19].

#### 2.5.4. Temperature Measurements

Dual-mode temperature assessments were conducted. Axillary temperature was recorded using Philips thermometer probe to provide systemic temperature readings. Additionally, regional (cutaneous from the finger) temperature was evaluated using the automated capillary refill time (aCRT) device with an embedded IR thermal sensor MLX90632 (Melexis NV, Tessenderlo, Belgium) at each stage, which aided in assessing localized changes in perfusion.

#### 2.5.5. Serum Lactate Levels

Serum lactate levels, indicative of metabolic status and tissue perfusion, were analyzed at two key stages: the start (T1) and the end (T4) of the study. These analyses were performed using a whole blood gas analyzer (GEM Premier 5000 blood gas testing system, Werfen, Barcelona, Spain) on arterial blood samples. This approach facilitated the assessment of resuscitation efficacy and the detection of potential tissue hypoperfusion.

### 2.6. Statistical Analysis

Due to the relatively small sample size, nonparametric tests were used in this study. Data were averaged for each stage (T1–T4) and analyzed, comparing the absolute values between the viral and Bacterial groups at each stage. Descriptive statistics were used to present the data as mean ± standard deviation (SD). The Mann–Whitney U test was employed to compare the Bacterial and COVID-19 groups in each phase of the protocol. For within-group comparisons between different protocol stages, the Friedman Repeated Measures Analysis of Variance on Ranks was used, followed by the Student–Newman–Keuls Method for post hoc analysis. Changes in lactate levels within the Bacterial and viral groups at different stages were analyzed using the Wilcoxon Signed-Rank Test. A *p*-value of <0.005 was considered significant. SPSS version 26.0 (IBM Corp., Armonk, NY, USA) was used for statistical analyses.

## 3. Results

### 3.1. Patient Characteristics and Demographic Data

In this single-center prospective study, the initial cohort consisted of 20 patients with ARDS or pneumonia requiring intensive care, split into two diagnostic groups: COVID-19 and Bacterial Septic Shock. The groups were comparable in terms of age, weight, and Body Mass Index (BMI), ensuring homogeneity between groups at the outset (see Table 1).

### 3.2. Clinical Characteristics and Medication

In this single-center prospective study, distinct patterns were observed in the clinical and pharmacological management of patients between the two diagnostic groups: COVID-19 and Bacterial Septic Shock. The average SOFA score on admission was lower in the COVID-19 group (4.80 ± 1.93) compared to the Bacterial group (8.10 ± 2.77), indicating less severe illness in the former group. The COVID-19 group required a lower noradrenaline dose (0.08 ± 0.06 mcg/kg/min) compared to the Bacterial group (0.22 ± 0.17 mcg/kg/min). However, the COVID-19 group required more midazolam (1.26 ± 0.34 mcg/kg/min) compared to the Bacterial group (0.83 ± 0.16 mcg/kg/min), indicating a greater need for sedation in the COVID-19 group. Furthermore, the COVID-19 group received more fentanyl (0.03 ± 0.01 mcg/kg/min) compared to the Bacterial group (0.01 ± 0.00 mcg/kg/min), pointing to a higher requirement for analgesia in the COVID-19 group.

### 3.3. Baseline Hemodynamic and Microcirculatory Parameters

At baseline (T1), the cohort was divided into two groups: COVID-19 and Bacterial. Several differences and similarities were observed between the groups.

**Figure 1:**
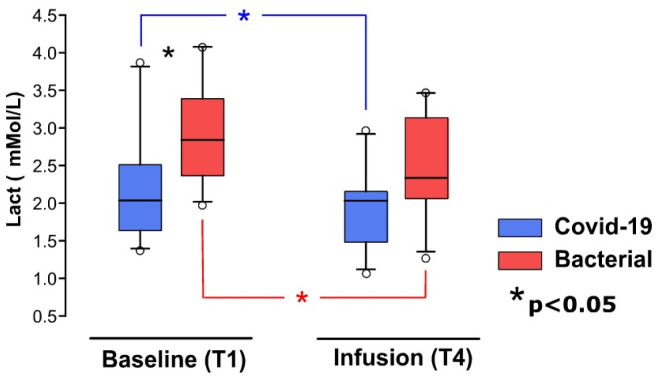
Comparison of blood lactate levels in patients with COVID-19 and Bacterial Septic Shock during the baseline and fluid infusion. Significant differences (*p* < 0.05) are denoted by asterisks: black for COVID-19 and Bacterial groups at same stage, blue for the COVID-19 group across different stages, and red for the Bacterial group across different stages.

In terms of metabolic response, the Bacterial group exhibited a higher initial serum lactate level (Bacterial: 2.91 ± 0.70 mM/L; COVID-19: 2.23 ± 0.77 mM/L) (see Figure 1). However, both groups showed comparable results in systemic hemodynamics, characterized by the Mean Arterial Pressure (MAP) (COVID-19: 78.37 ± 11.03 mmHg; Bacterial: 79.71 ± 6.42 mmHg).

Interestingly, no significant difference was observed between the patient groups in both the manually and automatically measured capillary refill time (CRT). The manually measured CRT (mCRT) was 2.28 ± 0.78 s for the COVID-19 group vs. 2.49 ± 0.66 s for the Bacterial group, and the automatically measured CRT (aCRT) was 1.93 ± 0.77 s for the COVID-19 group vs. 2.29 ± 0.85 s for the Bacterial group.

**Figure 2:**
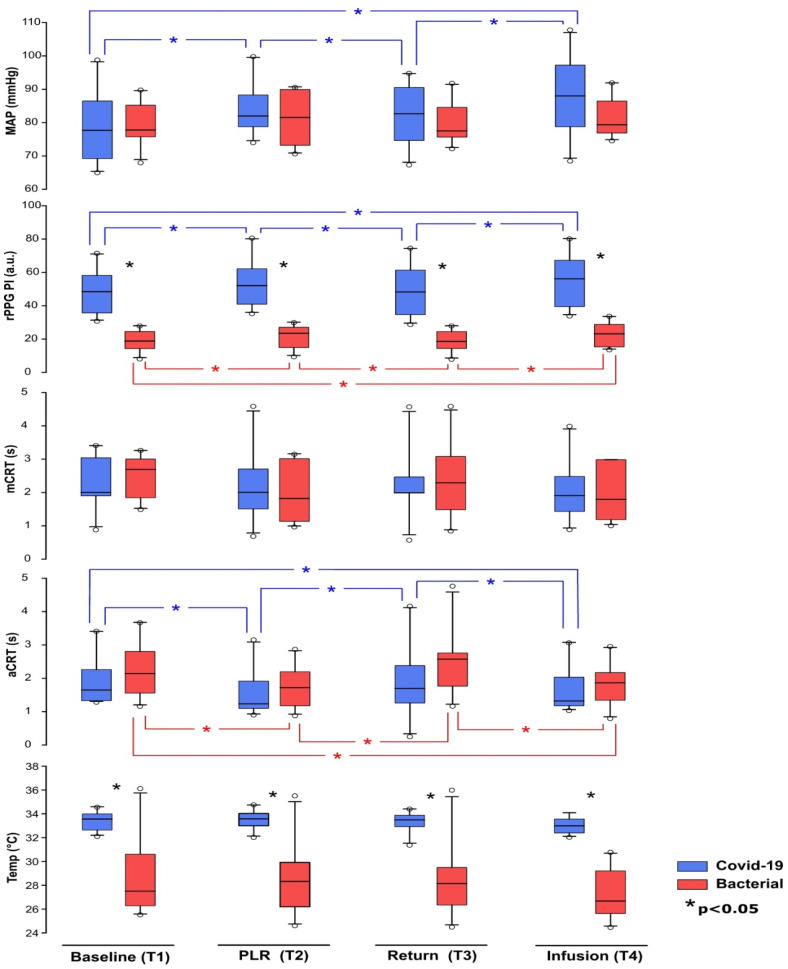
Comparison of physiological parameters of patients with COVID-19 and Bacterial Septic Shock across different protocol stages. Significant differences (*p* < 0.05) are denoted by asterisks: black for COVID-19 and Bacterial groups at same stage, blue for COVID-19 group across different stages, and red for Bacterial group across different stages.

The COVID-19 group demonstrated a higher mean relative perfusion index (rPPG) (COVID-19: 48.11 ± 13.27 a.u.; Bacterial: 18.68 ± 6.34 a.u.). The local temperature measurements differed significantly between the groups, with the COVID-19 group having a higher mean local temperature (COVID-19: 33.38 ± 0.79 °C; Bacterial: 28.51 ± 3.32 °C). However, the axillary temperatures did not significantly differ between the COVID-19 group (38.50 ± 0.34 °C) and the Bacterial group (38.01 ± 0.76 °C). A graphic representation of the results is provided in Figure 2.

### 3.4. Comparisons of Intervention Responses between Groups

#### 3.4.1. COVID-19 Group Intervention Response

In the COVID-19 group, the Passive Leg Raising Test (PLRT) maneuver elicited significant hemodynamic changes. An increase in the Mean Arterial Pressure (MAP) was observed from baseline T1 to T2 (COVID-19: 78.37 ± 11.03 mmHg to 84.13 ± 8.06 mmHg), indicating an initial positive systemic hemodynamic response to fluid. However, this response was transient, as the MAP returned towards baseline levels upon the cessation of the PLRT at T3 (82.37 ± 8.68 mmHg), with a subsequent increase after fluid administration at T4 (87.47 ± 11.83 mmHg).

The relative perfusion index (rPPG) data mirrored this transient change, with an increase at T2 (53.64 ± 14.54 a.u.), recovery to almost the baseline value at T3 (49.31 ± 15.01 a.u.), and a substantial elevation after fluid infusion at T4 (55.30 ± 15.79 a.u.).

Interestingly, the automatically measured capillary refill time (aCRT) but not the manually measured CRT (mCRT) statistically significantly changed during different protocol stages, reflecting the pattern of cutaneous perfusion (rPPG) and arterial pressure (MAP) changes. There was a decline from baseline T1 to T2 (1.93 ± 0.77 s to 1.58 ± 0.71 s), and it nearly returned towards the baseline level upon the cessation of the PLRT at T3 (1.92 ± 1.16 s) with a subsequent increase upon fluid infusion at T4 (1.68 ± 0.76 s).

Noteworthy was the relative stability of the local cutaneous temperature during the entire protocol course (T1: 33.38 ± 0.79 °C; T4: 33.01 ± 0.71 °C). These findings provide a comprehensive understanding of the COVID-19 group’s response to the intervention.

#### 3.4.2. Bacterial Group Intervention Response

The Bacterial Septic Shock group demonstrated a distinct response pattern throughout the stages of the protocol, which included the Passive Leg Raising Test (PLRT) and a fluid infusion stage.

While no statistically significant changes were observed in Mean Arterial Pressure (MAP), temperature, and manually detected capillary refill time (mCRT) at any stage of the protocol, the mean values indicated a similar trend to the COVID-19 group.

The most notable and statistically significant differences were observed in the variables characterizing microcirculation: the cutaneous perfusion index (rPPG) and the automatically detected capillary refill time (aCRT).

For rPPG, the mean value increased from baseline T1 to during the PLRT at T2 (Bacterial: 18.68 ± 6.34 a.u. to 21.08 ± 6.90 a.u.). Following the return of the leg to its initial position at T3, the mean rPPG value decreased slightly (18.55 ± 6.31 a.u.). However, following fluid infusion at T4, the mean rPPG value increased (22.49 ± 6.97 a.u.), suggesting a sustained impact on microcirculation.

In terms of aCRT, the mean value decreased from baseline T1 to during the PLRT at T2 (Bacterial: 2.29 ± 0.85 s to 1.76 ± 0.63 s). Upon the return of the leg to its initial position at T3, the mean aCRT value increased (2.53 ± 0.97 s). Following fluid infusion at T4, the mean aCRT value decreased (1.84 ± 0.64 s). Noteworthy was the relative stability of the temperature during the entire protocol course.

The comparative trend in responses between groups is depicted in Figure 1. The comprehensive results and statistics of this study are detailed in the tables provided in the Supplementary Materials.

## 4. Discussion

Our findings support the hypothesis that remote photoplethysmography (rPPG) and automated capillary refill time (aCRT) techniques are effective in evaluating alterations in peripheral perfusion during fluid resuscitation in patients with septic shock. The perfusion index (PI) measured by rPPG demonstrated higher sensitivity compared to the aCRT. Notably, the PI values significantly differed between the COVID-19 and Bacterial groups, whereas no significant differences were observed in the aCRT parameters between these groups. In our study, we observed changes in microcirculation between the COVID-19 and Bacterial Septic Shock groups, which could be explained by differences in pathophysiological mechanisms. COVID-19 is a unique viral infection with distinct pathophysiological features, particularly in its impact on the coagulatory and inflammatory systems. Our study aimed to compare the microcirculatory effects of bacterial and viral sepsis, with COVID-19 serving as a representative for viral sepsis given its relevance during the pandemic. It is well recognized that COVID-19-induced coagulopathy differs from other viral infections as it is associated with more frequent venous thromboembolism and arterial thrombosis. This contrasts with bacterial sepsis, which typically involves systemic hypercoagulation and suppressed fibrinolysis. COVID-19 involves both direct viral invasion of endothelial cells through ACE2 receptors and indirect damage via cytokine storms, contributing to endothelial dysfunction and microvascular injury [20,21]. Despite these distinctions, our study focused on the broader, shared mechanisms of microcirculatory dysfunction in both viral and bacterial sepsis. Sepsis, regardless of its cause, leads to common microcirculatory disturbances, such as endothelial cell damage, glycocalyx degradation, leukocyte adhesion, and microthrombus formation [22]

Our study results indicate significant differences between groups in terms of noradrenaline consumption, local skin temperature, and serum lactate levels. These differences may be attributed to more pronounced microcirculatory dysfunction in bacterial sepsis compared to COVID-19-associated sepsis.

Previous studies have demonstrated that patients with cold skin on their arms and legs exhibit lower central venous saturation and higher lactate levels [11,13]. The research conducted by the Guilherme Martins de Souza group reported similar findings, showing that patient with COVID-19 have a higher Peripheral Perfusion Index (PPI) than patients without COVID-19, as detected by an infrared camera [23]. The authors suggest that these findings may be related to fewer functional alterations in the microcirculation in COVID-19, indicating that microvascular reactivity dysfunction is not the primary mechanism in the pathophysiology of COVID-19. The research by Matthijs Kox et al. demonstrates that circulating cytokine levels are lower in patients with COVID-19 compared to those with bacterial sepsis [24]. Although COVID-19-associated sepsis exhibits unique characteristics, particularly in coagulopathy and immune response, it shares critical pathways of microvascular dysfunction with other viral sepsis forms.

In our study, patients with COVID-19 required significantly higher doses of midazolam and fentanyl for sedation, aligning with the findings of Kapp et al. [25], who reported that patients with COVID-19 with ARDS needed up to three times more sedatives compared to patients without COVID-19. Several factors likely contribute to this increased need, including the use of neuromuscular blocking agents (NMBAs), which often require deeper sedation to prevent awareness under paralysis, as well as high fevers and increased ventilatory drive in patients with COVID-19, exacerbating ventilator dyssynchrony. Additionally, the inflammatory and hypermetabolic state in severe COVID-19 may contribute to increased physical discomfort and ventilatory challenges, further raising sedation and analgesia requirements.

We observed an increase in the perfusion index during the Passive Leg Raising Test in both groups. This suggests that the perfusion index could serve as a sensitive indicator of fluid responsiveness in patients with septic shock, potentially enabling a reduction in the duration of negative fluid balance and the risk of fluid overload. J.L. Vincent, in the Annual Update in Intensive Care and Emergency Medicine [2], highlighted that the need for fluid administration in response to hypovolemia can be assessed at the bedside by observing a reduced functional capillary density (FCD) and perfused vessel density, which measure diffusive capacity, as well as microvascular flow index or RBC velocity, which indicate the convective capacity of microcirculation [2]. Furthermore, Duranteau et al. demonstrated that both passive leg raising (PLR) and volume expansion (VE) induced significant improvements in sublingual microcirculation in preload-dependent patients with septic shock [8].

We did not demonstrate a significant difference between groups when assessing the manual capillary refill time (mCRT). However, when using the automated capillary refill time (aCRT), differences were observed at each PLRT step between the two groups, suggesting that the aCRT is more sensitive than the mCRT. This observation, coupled with the small number of study participants, underscores the potential for the aCRT to provide more reliable measurements. Toll John R, Henricson J, and Anderson CD et al. found that the naked-eye-assessed capillary refill time exhibits poor reproducibility, even among the same observers, and significantly differs from the objectively measured capillary refill time [26]. Our study demonstrates that the rPPG and aCRT have the potential to provide non-invasive, real-time assessments of microcirculatory function, offering additional insights into a patient’s hemodynamic status. This is particularly relevant in sepsis, where restoring microcirculation is crucial to preventing tissue hypoxia and organ dysfunction. The integration of these novel techniques into routine clinical practice could have several important implications.

One such implication is enhanced monitoring of microcirculation. Currently, most intensive care units (ICUs) rely on systemic hemodynamic parameters such as the Mean Arterial Pressure (MAP) and serum lactate levels to guide resuscitation. However, these markers do not always reflect the state of the microcirculation, which can remain impaired even when the systemic parameters are normalized. By incorporating the rPPG and aCRT into routine monitoring, clinicians would have access to real-time, non-invasive indicators of peripheral perfusion, allowing for more precise guidance of fluid resuscitation and vasopressor therapy.

Another important implication is the early detection of microcirculatory dysfunction. The ability of rPPG to detect subtle changes in the perfusion index (PI) and the objective, automated measurements provided by the aCRT could enable earlier detection of microcirculatory dysfunction, which might not be immediately apparent using traditional methods. This could facilitate earlier interventions aimed at restoring adequate tissue perfusion, potentially improving outcomes in patients with septic shock.

Additionally, these techniques could help reduce the risk of fluid overload. One of the major challenges in managing patients with sepsis is determining the appropriate volume of fluid resuscitation. Over-resuscitation can lead to fluid overload, which is associated with increased morbidity and mortality. By using the rPPG and aCRT to more accurately assess microcirculatory responses to fluid challenges, such as Passive Leg Raising Tests, clinicians could tailor fluid therapy more precisely, reducing the risk of fluid overload while ensuring adequate perfusion.

Finally, improved resuscitation strategies could be developed. In line with the findings of studies like the ANDROMEDA shock trial, which suggested the benefit of perfusion-guided resuscitation strategies, our results indicate that the rPPG and aCRT could be valuable additions to the toolkit used to guide sepsis management. These techniques could complement existing parameters such as the capillary refill time (CRT) and lactate levels, providing a more comprehensive understanding of a patient’s perfusion status.

### Limitations of the Study

The findings of this study should be interpreted with caution due to several inherent limitations. However, these limitations are consistent with the preliminary nature of this investigation and do not diminish the study’s contributions to the understanding of microcirculatory dynamics in patients with sepsis.

Firstly, the small sample size (20 patients) is a key limitation, reducing the power to detect more subtle differences between bacterial- and COVID-19-associated sepsis. Small sample sizes are a common limitation in exploratory studies focused on microcirculation, such as those conducted by Dubin et al. (2010) [27], Edul et al. (2014) [28], Sadaka et al. (2011) [29], and Damiani et al. (2015) [30], which have similarly included 20–22 patients. While our sample size may limit the generalizability of the findings, this study was designed as a pilot investigation to assess the feasibility of using novel techniques—namely remote photoplethysmography (rPPG) and the automated capillary refill time (aCRT)—to monitor microcirculatory changes in a critical care setting. The results provide initial insights into the distinct microcirculatory responses between bacterial and viral etiologies of sepsis. To mitigate the impact of the small sample size, we employed nonparametric statistical tests, which are robust for handling smaller datasets. Future studies with larger cohorts will be necessary to confirm these initial observations and explore more microcirculatory differences.

Secondly, the single-center design of this study limits the external validity and generalizability of the results to other healthcare settings and patient populations. Institutional differences in ICU protocols, patient management, and local demographics could potentially influence the outcomes if this study was conducted in different centers. Nevertheless, the single-center design allowed for the uniform application of inclusion criteria, standardized measurement protocols, and consistent patient care. This consistency reduced potential variability, thereby enhancing the internal validity of the study. Although the results may not be directly transferable to all settings, they provide a controlled proof of concept that can serve as a foundation for future multicenter studies, which will be critical for validating and expanding upon these findings.

The subjectivity inherent in manual capillary refill time (CRT) measurements represents another limitation of this study. The manual CRT is dependent on the observer’s technique, environmental factors such as lighting, and individual interpretation, all of which introduce variability [26]. To minimize this bias, we employed a standardized protocol, ensured that all manual measurements were performed by a single trained observer, and averaged five measurements per time point to increase reliability. Moreover, we complemented manual CRT with automated CRT (aCRT), an objective measurement method that eliminates observer bias by using consistent pressure and optical sensors to detect the reperfusion upon the blanching. The use of aCRT in conjunction with manual CRT allowed us to compare results and demonstrated that aCRT provided more consistent data across time points and groups. While manual CRT remains widely used in clinical practice, our findings suggest that automated techniques, such as aCRT, offer a more reproducible and reliable alternative, particularly for future research and clinical applications.

Another limitation concerns the clinical significance of the observed microcirculatory changes. While we detected microcirculatory differences between bacterial and viral sepsis using rPPG and aCRT, it remains uncertain whether these changes directly impact clinical outcomes such as survival, organ dysfunction, or recovery trajectories. The relationship between microcirculatory improvement and clinical benefit remains unclear, and, to date, there is a lack of prospective trials specifically targeting microcirculatory endpoints in the resuscitation of patients with sepsis or COVID-19. Our study provides important preliminary data on the feasibility of the non-invasive monitoring of microcirculation, but future research must focus on establishing whether the observed changes translate into improved patient outcomes.

Lastly, as with all observational studies, the potential for residual confounding cannot be entirely excluded. Despite employing a rigorous statistical analysis and controlling for known confounders, there may be unmeasured variables influencing the results. Nonetheless, by using non-invasive, objective tools such as aCRT and rPPG, we minimized the variability introduced by manual methods and enhanced the reliability of our measurements. Future studies should aim to further reduce confounding factors by incorporating multicenter designs and controlling for additional potential confounders.

While this study has several limitations, it provides valuable pilot data on the use of novel, non-invasive techniques for assessing microcirculatory function in sepsis. These findings offer a foundation for larger, multicenter studies that will be essential to confirm and extend the observed differences in microcirculation between bacterial and viral sepsis.

## 5. Conclusions

In managing patients with sepsis in intensive care, the monitoring of microcirculation is of paramount importance in infusion therapy. Our study highlights the potential of remote photoplethysmography (PPG) and the automated capillary refill time (aCRT) as tools for this purpose. These techniques can be used in conjunction with routine parameters, such as lactate levels and systemic hemodynamic parameters, to provide a comprehensive assessment of a patient’s condition. However, the full realization of PPG and aCRT in clinical settings is presently limited, and further extensive research which includes enhancing the PPG signal quality and improving the measurement methodology are required. In conclusion, our findings underscore the potential of PPG and aCRT as cost-effective and non-invasive techniques for the assessment of microcirculation, contributing to improved sepsis management in intensive care in the future.

## Figures and Tables

**Table 1:** Patient demographics. The table displays the mean values ± standard deviations for the COVID-19 (n = 10) and Bacterial Septic Shock groups (n = 10). Statistically significant differences (*p* < 0.005) are indicated by an asterisk (*).

Patient Characteristics and Treatment Parameters	COVID-19 (n = 10)	Bacterial Septic Shock (n = 10)
Age, years	59.6 ± 9.90	64.7 ± 11.41
Male gender, n (%)	65	59
Height, cm	168.6 ± 10.32	177.2 ± 5.53
Weight, kg	78.9 ± 17.15	85.7 ± 18.75
BMI, kg/m^2^	27.84 ± 5.67	27.18 ± 5.15
SOFA, points	4.80 ±1.93 *	8.10 ± 2.77
Noradrenaline, mcg/kg/min	0.08 ± 0.06 *	0.22 ± 0.17
Midazolam, mcg/kg/min	1.26 ± 0.34 *	0.83 ± 0.16
Fentanyl, mcg/kg/min	0.03 ± 0.01 *	0.01 ± 0.00

## Data Availability

The original contributions presented in this study are included in the article’s material; further inquiries can be directed to the corresponding author(s).

## Supplementary Materials

The following supporting information can be downloaded at https://www.mdpi.com/article/10.3390/medicina60101680/s1, Table S1: Demographic data for viral Group Patients. Table S2: Demographic data for bacterial Group Patients. Table S3: mean arterial blood pressure (MAP) (mmHg) Statistics for Bacterial Group at each time interval: passive leg raising test (PLRT) T1 (baseline), T2 (during PLRT), T3 (post-PLRT), and T4 after fluid expansion. Table S4: automated capillary refill time (aCRT) ( seconds (s) ) Statistics for Bacterial Group at each time interval: passive leg raising test (PLRT) T1 (baseline), T2 (during PLRT), T3 (post-PLRT), and T4 after fluid expansion. Table S5: manual capillary refill time (mCRT) (s) Statistics for Bacterial Group at each time interval: passive leg raising test (PLRT) T1 (baseline), T2 (during PLRT), T3 (post-PLRT), and T4 after fluid expansion. Table S6: Local skin temperature °C Statistics for Bacterial Group at each time interval: passive leg raising test (PLRT) T1 (baseline), T2 (during PLRT), T3 (post-PLRT), and T4 after fluid expansion. Table S7: Serum lactate levels (mM/L) Statistics for Bacterial Group during T1 (baseline), and T4 after fluid expansion. Table S8: mean arterial blood pressure (MAP) (mmHg) Statistics for viral group at each time interval: passive leg raising test (PLRT) T1 (baseline), T2 (during PLRT), T3 (post-PLRT), and T4 after fluid expansion. Table S9: Photoplethysmography (PPG) (a.u.) Statistics for viral group at each time interval: passive leg raising test (PLRT) T1 (baseline), T2 (during PLRT), T3 (post-PLRT), and T4 after fluid expansion. Table S10: automated capillary refill time (aCRT) (seconds (s)) Statistics for viral group at each time interval: passive leg raising test (PLRT) T1 (baseline), T2 (during PLRT), T3 (post-PLRT), and T4 after fluid expansion. Table S11: manual capillary refill time (mCRT) (seconds (s)) statistics for viral group at each time interval: passive leg raising test (PLRT) T1 (baseline), T2 (during PLRT), T3 (post-PLRT), and T4 after fluid expansion. Table S12: serum lactate levels (mM/L) statistics for viral group at each time interval: passive leg raising test (PLRT) T1 (baseline), T2 (during PLRT), T3 (post-PLRT), and T4 after fluid expansion. Table S13. Local skin temperature °C Statistics for viral group at each time interval: passive leg raising test (PLRT) T1 (baseline), T2 (during PLRT), T3 (post-PLRT), and T4 after fluid expansion.

## References

[1] Fleischmann-Struzek C., Goldfarb D.M., Schlattmann P., Schlapbach L.J., Reinhart K., Kissoon N. (2018). The global burden of paediatric and neonatal sepsis: A systematic review. Lancet Respir. Med..

[2] Vincent J.-L. (2023). Annual Update in Intensive Care and Emergency Medicine.

[3] Evans L., Rhodes A., Alhazzani W., Antonelli M., Coopersmith C.M., French C., MacHado F.R., McIntyre L., Ostermann M., Prescott H.C. (2021). Surviving Sepsis Campaign: International Guidelines for Management of Sepsis and Septic Shock 2021. Crit. Care Med..

[4] WHO (2020). Global Report on the Epidemiology and Burden of Sepsis: Current Evidence, Identifying Gaps and Future Directions.

[5] Beltrán-García J., Osca-Verdegal R., Pallardó F.V., Ferreres J., Rodríguez M., Mulet S., Ferrando-Sánchez C., Carbonell N., García-Giménez J.L. (2020). Sepsis and Coronavirus Disease 2019: Common Features and Anti-Inflammatory Therapeutic Approaches. Crit. Care Med..

[6] Kattan E., Ibarra-Estrada M., Ospina-Tascón G., Hernández G. (2023). Perspectives on peripheral perfusion assessment. Curr. Opin. Crit. Care.

[7] Hernández G., Bakker J. (2019). Perspectives on perfusion monitoring in septic shock after the ANDROMEDA-SHOCK trial. Med. Intensiv..

[8] Duranteau J., De Backer D., Donadello K., Shapiro N.I., Hutchings S.D., Rovas A., Legrand M., Harrois A., Ince C. (2023). The future of intensive care: The study of the microcirculation will help to guide our therapies. Crit. Care.

[9] Neto A.S., Pereira V.G.M., Manetta J.A., Espósito D.C., Schultz M.J. (2014). Association between static and dynamic thenar near-infrared spectroscopy and mortality in patients with sepsis: A systematic review and meta-analysis. J. Trauma Acute Care Surg..

[10] Yusuf G.T., Wong A., Rao D., Tee A., Fang C., Sidhu P.S. (2022). The use of contrast-enhanced ultrasound in COVID-19 lung imaging. J. Ultrasound.

[11] Hariri G., Joffre J., Leblanc G., Bonsey M., Lavillegrand J.R., Urbina T., Guidet B., Maury E., Bakker J., Ait-Oufella H. (2019). Narrative review: Clinical assessment of peripheral tissue perfusion in septic shock. Ann. Intensive Care.

[12] Hernández G., Cavalcanti A.B., Ospina-Tascón G., Zampieri F.G., Dubin A., Hurtado F.J., Friedman G., Castro R., Alegría L., Cecconi M. (2018). Early goal-directed therapy using a physiological holistic view: The ANDROMEDA-SHOCK-a randomized controlled trial. Ann. Intensive Care.

[13] Kaplan L.J., McPartland K., Santora T.A., Trooskin S.Z. (2001). Start with a subjective assessment of skin temperature to identify hypoperfusion in intensive care unit patients. J. Trauma.

[14] Monnet X., Teboul J.L. (2015). Passive leg raising: Five rules, not a drop of fluid!. Crit. Care.

[15] Mallat J., Fischer M.O., Granier M., Vinsonneau C., Jonard M., Mahjoub Y., Baghdadi F.A., Préau S., Poher F., Rebet O. (2022). Passive leg raising-induced changes in pulse pressure variation to assess fluid responsiveness in mechanically ventilated patients: A multicentre prospective observational study. Br. J. Anaesth..

[16] Fleming S., Gill P., Jones C., Taylor J.A., Van Den Bruel A., Heneghan C., Roberts N., Thompson M. (2015). The Diagnostic Value of Capillary Refill Time for Detecting Serious Illness in Children: A Systematic Review and Meta-Analysis. PLoS ONE.

[17] Marcinkevics Z., Laksa E., Rubenis O., Blumfelde M. Reliability of automated optical determination of capillary refill time. Proceedings of the Biophotonics Congress 2021, Bio-Optics: Design and Application 2021.

[18] Wu T., Blazek V., Schmitt H.J. (2000). Photoplethysmography imaging: A new noninvasive and noncontact method for mapping of the dermal perfusion changes. Optical Techniques and Instrumentation for the Measurement of Blood Composition, Structure, and Dynamics.

[19] Hagar H., Church S., Mandadi G., Pulley D., Kurz A. (2004). The perfusion index measured by a pulse oximeter indicates pain stimuli in anesthetized volunteers. Anesthesiology.

[20] Hawiger J., Veach R.A., Zienkiewicz J. (2015). New paradigms in sepsis: From prevention to protection of failing microcirculation. J. Thromb. Haemost..

[21] Largman-Chalamish M., Wasserman A., Silberman A., Levinson T., Ritter O., Berliner S., Zeltser D., Shapira I., Rogowski O., Shenhar-Tsarfaty S. (2022). Differentiating between bacterial and viral infections by estimated CRP velocity. PLoS ONE.

[22] Massey M.J., Hou P.C., Filbin M., Wang H., Ngo L., Huang D.T., Aird W.C., Novack V., Trzeciak S., Yealy D.M. (2018). Microcirculatory perfusion disturbances in septic shock: Results from the ProCESS trial. Crit. Care.

[23] de Souza G.M., Galindo V.B., da Rocha D.L., Vianna F.S.L., Chaves R.C.F., Malossi C.D., Vieira A.M., Midega T.D., Cavalcanti F.M., Assunção M.S.C. (2024). Assessment of Peripheral Perfusion in Severe Acute Respiratory Syndrome Coronavirus 2 (SARS-CoV-2) Infection: An Exploratory Analysis with Near-Infrared Spectroscopy. Arch. Microbiol. Immunol..

[24] Kox M., Waalders N.J.B., Kooistra E.J., Gerretsen J., Pickkers P. (2020). Cytokine Levels in Critically Ill Patients With COVID-19 and Other Conditions. JAMA.

[25] Kapp C.M., Zaeh S., Niedermeyer S., Punjabi N.M., Siddharthan T., Damarla M. (2020). The Use of Analgesia and Sedation in Mechanically Ventilated Patients With COVID-19 Acute Respiratory Distress Syndrome. Anesth. Analg..

[26] Toll John R., Henricson J., Anderson C.D., Björk Wilhelms D. (2019). Man versus machine: Comparison of naked-eye estimation and quantified capillary refill. Emerg. Med. J..

[27] Dubin A., Pozo M.O., Casabella C.A., Murias G., Pálizas F., Moseinco M.C., Kanoore Edul V.S., Pálizas F., Estenssoro E., Ince C. (2010). Comparison of 6% hydroxyethyl starch 130/0.4 and saline solution for resuscitation of the microcirculation during the early goal-directed therapy of septic patients. J. Crit. Care.

[28] Edul V.S.K., Ince C., Navarro N., Previgliano L., Risso-Vazquez A., Rubatto P.N., Dubin A. (2014). Dissociation between sublingual and gut microcirculation in the response to a fluid challenge in postoperative patients with abdominal sepsis. Ann. Intensive Care.

[29] Sadaka F., Aggu-Sher R., Krause K., O’Brien J., Armbrecht E.S., Taylor R.W. (2011). The effect of red blood cell transfusion on tissue oxygenation and microcirculation in severe septic patients. Ann. Intensive Care.

[30] Damiani E., Adrario E., Luchetti M.M., Scorcella C., Carsetti A., Mininno N., Pierantozzi S., Principi T., Strovegli D., Bencivenga R. (2015). Plasma free hemoglobin and microcirculatory response to fresh or old blood transfusions in sepsis. PLoS ONE.

