# Supplementary material for: Quantitative Evaluation of Microcirculatory Alterations in Patients with COVID-19 and Bacterial Septic Shock through Remote Photoplethysmography and Automated Capillary Refill Time Analysis
Source: Medicina (Kaunas). 2024 Oct 14;60(10):1680. doi: 10.3390/medicina60101680 (PMC11509756; doi:10.3390/medicina60101680)
Supplement: Supplementary file 1 [file medicina-60-01680-s001.zip › Tables fin .pdf]

**Statistics**	**Age (years)**	**Height (cm)**	**Wight (kg)**	**BMI** kg/m²	**Noradrenalin** mcg/kg /min	**Midazolam** mcg/kg /min	**Fentanyl** mcg/kg /min	**SOFA**	**Temp Axillary** °C
Mean	59.6	168.6	78.9	27.84	0.08	1.26	0.03	4.8	38.5
Std Dev	9.9	10.32	17.15	5.67	0.06	0.34	0.01	1.93	0.34
Median	60.0	170.5	75.0	25.62	0.07	1.26	0.03	4.0	38.5
IQR	8.0	14.75	17.25	6.66	0.1	0.37	0.01	2.75	0.38
95% C.I. Lower	52.52	161.22	66.63	23.79	0.04	1.02	0.02	3.42	38.26
95% C.I. Upper	66.68	175.98	91.17	31.9	0.12	1.5	0.03	6.18	38.74
n	10.0	10.0	10.0	10.0	10.0	10.0	10.0	10.0	10.0

Table S1: Demografic data for viral Group Patients

**Statistics**	**Age (years)**	**Height (cm)**	**Wight (kg)**	**BMI** kg/m²	**Noradrenalin** mcg/kg /min	**Midazolam** mcg/kg /min	**Fentanyl** mcg/kg /min	**SOFA**	**Temp Axillary** °C
Mean	64.7	177.2	85.7	27.18	0.22	0.83	0.01	8.1	38.01
Std Dev	11.41	5.53	18.75	5.15	0.17	0.16	0.0	2.77	0.76
Median	68.0	180.0	80.0	25.2	0.15	0.8	0.01	7.0	38.25
IQR	12.75	3.75	27.0	7.45	0.21	0.17	0.01	4.25	1.15
95% C.I. Lower	56.54	173.24	72.29	23.5	0.1	0.72	0.01	6.12	37.47
95% C.I. Upper	72.86	181.16	99.11	30.87	0.34	0.95	0.01	10.08	38.55
n	10.0	10.0	10.0	10.0	10.0	10.0	10.0	10.0	10.0

Table S2: Dempgrafic data for bacterial Group Patients

Bacterial septic shock group

Statistics	T1MAP_B<br>baseline	T2MAP_B<br>during PLRT	T3MAP_B<br>post-PLRT	T4MAP_B<br>after fluid expansion
Mean	79.71	81.66	80.07	81.21
Std Dev	6.42	8.12	6.16	5.89
Median	77.8	81.77	77.5	79.2
IQR	8.63	15.07	7.13	7.05
95% C.I. Lower	75.73	76.63	76.25	77.56
95% C.I. Upper	83.69	86.69	83.89	84.86

Table S3: mean arterial blood pressure (MAP) (mmHg) Statistics for Bacterial Group at each time interval: passive leg raising test (PLRT) T1 (baseline), T2 (during PLRT), T3 (post-PLRT), and T4 after fluid expansion.

Statistics	T1aCRT baseline	T2aCRT_B during PLRT	T3aCRT_B post-PLRT	T4aCRT_B after fluid expansion
Mean	2.29	1.76	2.53	1.84
Std Dev	0.85	0.63	0.97	0.64
Median	2.18	1.73	2.59	1.89
IQR	0.88	0.95	0.77	0.64
95% C.I. Lower	1.77	1.37	1.93	1.44
95% C.I. Upper	2.82	2.16	3.14	2.23

Table S4: automated capillary refill time (aCRT) (seconds (s)) Statistics for Bacterial Group at each time interval: passive leg raising test (PLRT) T1 (baseline), T2 (during PLRT), T3 (post-PLRT), and T4 after fluid expansion.

Statistics	T1mCRT_B Baseline	T2mCRT_B During PLRT	T3mCRT_B Post-PLRT	T4mCRT_B After fluid expansion
Mean	2.49	2.06	2.37	2.03
Std Dev	0.66	0.91	1.14	0.88
Median	2.71	1.83	2.28	1.81
IQR	1.05	1.76	1.34	1.79
95% C.I. Lower	2.08	1.5	1.66	1.48
95% C.I. Upper	2.9	2.63	3.08	2.58

Table S5: manual capillary refill tiem (mCRT) (s) Statistics for Bacterial Group at each time interval: passive leg raising test (PLRT) T1 (baseline), T2 (during PLRT), T3 (post-PLRT), and T4 after fluid expansion.

Statistics	T1Temp_B baseline	T2Temp_B during PLRT	T3Temp_B post-PLRT	T4Temp_B after fluid expansion
Mean	28.51	28.42	28.43	27.24
Std Dev	3.32	3.08	3.14	2.13
Median	27.43	28.25	28.1	26.65
IQR	3.08	3.13	2.5	3.19
95% C.I. Lower	26.46	26.51	26.48	25.92
95% C.I. Upper	30.57	30.32	30.37	28.56

Table S6: Local skin temperature°C **Statistics for Bacterial Group at each time interval: passive leg raising test (PLRT) T1 (baseline), T2 (during PLRT), T3 (post-PLRT), and T4 after fluid expansion.**

**Statistics**	**T1lact_B**<br>baseline	**T4Lact_B**<br>after fluid expansion
Mean	2.91	2.48
Std Dev	0.7	0.67
Median	2.85	2.35
IQR	0.75	0.9
95% C.I. Lower	2.48	2.06
95% C.I. Upper	3.34	2.9

Table S7: Serum lactate levels (mM/L) Statistics for Bacterial Group during T1 (baseline), and T4 after fluid expansion.

Viral (COVID-19) group

**Statistics**	**T1MAP_V**	**T2MAP_V**	**T3MAP_V**	**T4MAP_V**
Mean	78.37	84.13	82.37	87.47
Std Dev	11.03	8.06	8.68	11.83
Median	77.68	81.98	82.66	88.0
IQR	13.85	6.8	12.12	15.5
95% C.I. Lower	71.53	79.13	76.99	80.14
95% C.I. Upper	85.21	89.12	87.75	94.8

Table S8: mean arterial blood pressure (MAP) (mmHg) Statistics for viral group at each time interval: passive leg raising test (PLRT) T1 (baseline), T2 (during PLRT), T3 (post-PLRT), and T4 after fluid expansion.

**Statistics**	**T1PPG _V**	**T2PPG_V**	**T3PPG _V**	**T4PPG_V**
Mean	48.11	53.64	49.31	55.3
Std Dev	13.27	14.54	15.01	15.79
Median	47.95	52.26	48.01	55.9
IQR	17.3	15.39	19.26	19.9
95% C.I. Lower	39.89	44.63	40.01	45.52
95% C.I. Upper	56.34	62.65	58.62	65.09

Table S9: Photoplethysmography (PPG) (a.u.) Statistics for viral group at each time interval: passive leg raising test (PLRT) T1 (baseline), T2 (during PLRT), T3 (post-PLRT), and T4 after fluid expansion.

Statistics	T1aCRT_V	T2aCRT_V	T3aCRT_V	T4aCRT_V
Mean	1.93	1.58	1.92	1.68
Std Dev	0.77	0.71	1.16	0.76
Median	1.67	1.27	1.72	1.34
IQR	0.54	0.55	0.58	0.47
95% C.I. Lower	1.46	1.14	1.2	1.21
95% C.I. Upper	2.41	2.01	2.65	2.14

Table S10: automated capillary refill time (aCRT) (seconds (s)) Statistics for viral group at each time interval: passive leg raising test (PLRT) T1 (baseline), T2 (during PLRT), T3 (post-PLRT), and T4 after fluid expansion.

Statistics	T1mCRT_V	T2mCRT_V	T3mCRT_V	T4mCRT_V
Mean	2.28	2.16	2.25	2.04
Std Dev	0.78	1.06	1.01	0.9
Median	2.0	2.0	2.0	1.9
IQR	0.92	0.9	0.22	0.67
95% C.I. Lower	1.79	1.5	1.62	1.48
95% C.I. Upper	2.76	2.82	2.88	2.6

Table S11: manual capillary refill time (mCRT) (seconds (s)) statistics for viral group at each time interval: passive leg raising test (PLRT) T1 (baseline), T2 (during PLRT), T3 (post-PLRT), and T4 after fluid expansion.

Statistic	T1lact _V	T4Lact_V
Mean	2.23	1.96
Std Dev	0.77	0.53
Median	2.05	2.05
IQR	0.5	0.53
95% C.I. Lower	1.75	1.63
95% C.I. Upper	2.71	2.29

Table S12: serum lactate levels (mM/L) statistics for viral group at each time interval: passive leg raising test (PLRT) T1 (baseline), T2 (during PLRT), T3 (post-PLRT), and T4 after fluid expansion.

Statistics	T1Temp_V	T2Temp_V	T3Temp_V	T4Temp_V
Mean	33.38	33.49	33.36	33.01
Std Dev	0.79	0.79	0.86	0.71
Median	33.5	33.56	33.53	33.0
IQR	1.03	0.88	0.75	0.9
95% C.I. Lower	32.89	33.0	32.82	32.58
95% C.I. Upper	33.87	33.98	33.89	33.45

Table S13. Local skin temperature°C **Statistics for viral group at each time interval: passive leg raising test (PLRT) T1 (baseline), T2 (during PLRT), T3 (post-PLRT), and T4 after fluid expansion.**
